# The Art of Counseling in the Treatment of Head and Neck Cancer: Exploratory Investigation among Perceptions of Health Professionals in Southern Italy

**DOI:** 10.3390/curroncol29090493

**Published:** 2022-08-31

**Authors:** Raffaele Addeo, Luca Pompella, Pasquale Vitale, Silvia Ileana Sara Fattoruso, Ilaria Di Giovanni, Francesco Perri, Michele Caraglia, Morena Fasano, Raffaele Arigliani

**Affiliations:** 1 U.O.C. Oncology “S. Giovanni di Dio” Hospital, ASLNA2NORD Via Pirozzi Frattamaggiore, 80027 Naples, Italy; 2 Division of Medical Oncology, Department of Precision Medicine, University of Campania “Luigi Vanvitelli”, 81100 Naples, Italy; 3 Head and Neck Medical Oncology Unit, Istituto Nazionale Tumori, IRCCS Fondazione G. Pascale, 80131 Naples, Italy; 4 Department of Precision Medicine, University of Campania “Luigi Vanvitelli”, 81100 Naples, Italy; 5 Biogem Scarl, Molecular Oncology and Precision Medicine Laboratory, Via Camporeale, 83031 Ariano Irpino, Italy; 6 School of Counselling, Italian Medical Research Institute, 82100 Benevento, Italy

**Keywords:** metastatic squamous cell carcinoma of the head and neck, counseling, open communication

## Abstract

(1) Background: Recurrent and/or metastatic patients with head and neck squamous cell carcinoma show a poor prognosis, which has not changed significantly in 30 years. Preserving quality of life is a primary goal for this subset of patients; (2) Methods: A group of 19 physicians working in South Italy and daily involved in head and neck cancer care took an anonymous online survey aimed at revealing the level of knowledge and the application of communication techniques in daily patient care; (3) Results: Several specialists, 18 out 19 (95%), considered that patient participation in therapeutic choices is mandatory. The main obstacles to complete and reciprocate communication still consist of lack of time and staff, but also in the need for greater organization, which goes beyond the multidisciplinary strategy already used; (4) Conclusions: A greater impulse to training and updating on issues related to counseling can improve communication between the different clinicians involved in the treatment plan.

## 1. Introduction

Head and neck (H&N) cancers are a complex and heterogeneous group of malignancies that require multifaceted treatment strategies and the input of several specialties. Despite the advances in multimodal treatments, the prognosis remains poor due to the high rate of advanced disease at diagnosis and the high rate of recurrence. At least 50% of patients with locally advanced disease are likely to develop locoregional or distant relapses within the first 2 years of treatment [1]. For this subset of patients, improved patient–physician communication is essential in the context of serious and life-limiting illnesses, with clear effects of good communication on quality of care and quality of life (QoL). In addition, an ethical mandate by which patients are involved and participate in informed decisions about their care is also essential [2]. In advanced H&N cancer, inadequate communication about prognosis and treatment choices is frequent [3] and is associated with unrealistic patient expectations about curability and with an aggressive treatment proposal that is not consistent with patient preferences [4,5]. Within the hospital, critical conversations typically do not occur or occur shortly before the start of the treatment program. Most advanced cancer patients want to be actively involved in their care and request open and sensitive conversations about quality of life, prognosis, and treatment choices [6]. Patient participation in treatment decision improves patient satisfaction and treatment adherence, positively influencing oncological care [7]. Multimodal treatment with a multidisciplinary team has become the standard option with a positive impact on patient assessment and management, improving the survival of stage IV patients [8,9]. However, this approach can represent a barrier to the participation of real patients, because values and preferences are not acknowledged during multidisciplinary discussion, which has been described in the health care field as ‘in absentia’ [10].

Clinicians often feel unprepared to have conversations that can include emotional reactions and address challenging side effects related to treatment. These difficulties are greater in the setting of patients who need palliative care, and are independent of the experience of the doctors [11]. In clinical practice, clinicians frequently think they are better communicators than their colleagues or their patients’ opinion [12]; however, it represents only an illusion and often communication is not happening. The discussion of controversial topics, such as treatment complications and lifestyle outcomes, should be aimed with particular attention to feelings. Patients with advanced H&N cancers prefer that clinicians discuss the topic and expect that they do [13]. Among patients with head and neck cancer, those awaiting the start of palliative chemotherapy are expected to have the highest degree of distress. Long periods of treatment, repeated hospitalization, and side effects of chemotherapy can affect the psychological status of these patients and influence communication with specialists. The major problem with communication is the illusion that it has occurred. Knowledge of strengths such as the involvement of a family member or patient autonomy and weaknesses such as caregiver disagreement or time and reimbursement constraints with health professionals can facilitate the development of patient-centered care [14]. The purpose of this observational study was to investigate the level of knowledge of counseling among clinicians involved in the treatment of H&N cancer and to highlight the barriers and aspects of the doctor–patient and doctor–family relationships that need improvement.

## 2. Materials and Methods

Data were collected between January 2020 and March 2020, using an anonymous online survey administered to 19 specialist physicians, through the SurveyMonkey ^®^ platform. The group of 19 physicians, working in South Italy and selected according to their daily involvement in H&N care, included oncologists and radiotherapists. We have included in the present study professional persons with consolidated experience in the specific field of at least 10 years All of them who had participated in a course on counseling dedicated to the patient with squamous cell carcinoma of the head and neck (SSCHN). The affiliations of all the participants are as follows: S. B., Radiotherapy Unit Asp di Siracusa (Sicily); B. S., Radiotherapy Unit INT Pascale Naples (Campania); A.C., Radiotherapy UNIT Centro Aktis Marano (Campania); M.C., Radiotherapy UNIT Acerra ASLNA2NORD (Campania); A. M., Di Grazia Humanitas Oncology Unit CCO Catania (Sicily); I. D., Radiotherapy UNIT Ospedale del Mare Naples (Campania); F. D., Radiotherapy Unit Cen-tro Morrone Caserta; M.F., Oncology Unit AOU Vanvitelli Naples (Campania); I F, Unit on Clinic Radiotherapy Macchiarella Palermo (Sicily); D G, Oncology Unit AO San Pio Benevento (Campania); M.G., Oncology Unit spedale Oncologico “A. Businco” Cagliari (Sardegna); F. P. and S.C., Oncologia Clinica Sperimentale Testa-Collo INT Pascale Naples (Campania); T.P., UO Radioterapia AO San Pio Benevento (Campania); N.R., Oncology Unit Policlinico Vittorio Emanuele Cata-nia (Sicily); G.R., Oncologist Ospedale Vito Fazzi di Lecce (Puglia); A.S., Oncologist Ospedale del Mare Naples (Campania); R S, Radiotherapy istituto DAM Nocera Inferiore (Campania); M. S., Oncology Unit Ospedale Giglio Cefalù (Sicily); and G. T., Oncology Unit Sant Anna e San Se-bastiano Caserta (Campania).Written informed consent was obtained from all participants. All the investigators returned the questionnaire (ratio of acceptance 100%), marking only with initials, and were therefore included in the analysis. This survey was specifically organized to investigate the value and level of knowledge of shared communication and counseling in the management of cancer patients (Table 1).

Seventeen questions, including 14 multiple choice questions, were submitted to the participants. The first part of the survey, points 1 through 6, investigated the state of the art shared with patient suffering from H&N cancers and the role of counseling. In the remaining 9 questions, we investigated the needs and critical issues that hinder and limit shared communication.

## 3. Results

All physicians, clinical oncologists, and radiotherapists specialized in the treatment of H&N cancer who attended a meeting on the counseling of H&N cancer patients in Naples during November 2019 returned the questionnaire (rate of acceptance 100%) and therefore were included in the analysis. Most of the respondents to the first question, 8 out of 19, (40%) considered clear and complete communication as the foundation that strengthens a therapeutic alliance, also pointing out the need to have organized and efficient healthcare personnel. To confirm these data, 12 out of 19 medical doctors (65%) considered that investigating patient and caregiver expectations for the current visit and facing every point during the examination represents a priority to make communication between patient and health care practitioner (HCP) stronger and more efficient (data from question two). However, only five (25%) specialists considered patient participation in the care pathway to be the main strength of their unit to pursue patient-focused care (Table 2).

However, most of the interviewees, responding to Question four, aimed to improve dialogue skills and patient management (Table 3).

However, replying to question five, most clinicians (10 out of 19) thought that overcrowding and lack of time represent the main weaknesses in seeking patient-focused care while doing daily work. Eight of nineteen physicians (40%) thought that staff and equipment shortages inhibit empathic communication with patients in daily practice (question six). Answering question eight, the lack of time represents the weakness of the unit to pursue a patient-focused care for 13 out 19 (70%) participants in the survey. Consequently, regarding question nine, 12 of the 19 (65%) doctors interviewed believed that the principal objectives not reached are: taking time listening to the patient more than talking, and the ability to investigate patient doubts and anxieties. Open multidisciplinary confrontation and dialogue skills with patients and caregivers represent an aspect that clinicians would like to improve during daily clinical practice, as evidenced by the responses provided in the questionnaire. Eleven out nineteen physicians (58%) have chosen this option in answering question ten. However, as requested in question one, only five (25%) specialists considered “open communication” to be the most relevant need for patients with metastatic and/or recurrent H&N cancer. Instead, the majority continue to consider as a priority the need for nutritional counseling and pain therapy. The counseling and effective communication with the multidisciplinary approach represented the most peculiar topics for almost all interviewees in relation to the treatment of H&N advanced cancer, as shown in the answers to question 12 of the questionnaire. Eleven (60%) respondents to question 11 continued to consider the multidisciplinary approach as the most relevant topic in H&N cancer therapy, but only a minority, 8 out 19, thought that this approach cannot be pursued without effective counseling and communication with the patient and their caregivers (question 13). Fourteen responders out of nineteen clinicians involved declared that they use counseling techniques more than enough in clinical practice in answering question 14. Fourteen out of nineteen (74%) of the interviewees considered themselves at least partially satisfied with their daily work in respect to the interaction with the patient.

## 4. Discussion

Squamous cell carcinoma of the head and neck remains a challenging clinical problem, with half a million new cases annually worldwide. Despite the recent development of new therapeutic options such as immunotherapy, patients with advanced disease still have low chances of being cured with current therapies [15].

Several studies and research have established that H&N cancer care provided through an integrated multidisciplinary team (MDT) approach determines improved patient outcome and better survival rates [7,16]. This innovative approach could increase efficiency in care delivery, reduce costs, and shorten hospital stay [17,18]. This core of the approach is represented by the exchange of information and dialogue between the various professionals involved in the treatment pathway.

In this setting, showing respect for the patient’s preferences and ensuring a better quality of life represent today the first aims that the doctor must pursue. This should pass through a shared communication obtained by listening to the preferences and needs of the patient [19]. Effective communication for cancer patients can meet information needs, reduce caregiver burden, improve physical and mental health, and promote intimacy. However, cancer patients and/or caregivers have different communication needs in terms of target, content, style, and communication timing. Data have suggested that communication preferences are related to factors such as demographics and ethnic origin [20].

In recent decades, attention has focused on efforts to improve doctor–patient communication, believing that such improvements would improve quality of care and patient outcomes [21,22]. Almost all specialists who participated in the survey confirmed that they are knowledgeable about the techniques of counseling; however, they claim to apply these counseling skills only partially, attributing the greatest difficulty to the limited time available in daily clinical practice.

All participants recognized the importance and priority of the multidisciplinary approach for the treatment of these tumors. However, they confirmed the presence of a series of obstacles that actually limit its application, including some difficulties in open communication. The dialogue between physicians and patients is the core of quality health care. It is essential that patient values are respected, and it is important to obtain patient preferences and goals [23].

The results of a recent study highlight the importance of exploring the thoughts and needs of patients, in order to enhance patient-centered care. Patients with head and neck cancer found it important to receive information on their life expectancy [24]. Professionals with better communication and interpersonal skills provide better support to their patients. The current productivity-oriented practice environment also presents barriers to effective communication; the experts involved in this observational survey confirmed the presence of several obstacles that slow the adequate development and application of counseling techniques, such as overcrowding and lack of time, but also internal conflicts and lack of appropriate knowledge about counseling. These original results were obtained on a representative but limited sample of medical oncologists and radiotherapists operating in some centers of Southern Italy, who are daily involved in the treatment of patients with SSCHN. The limited number of professionals involved in the present survey was due to the absence in some regions in Southern Italy of specialistic centers with a consolidated experience in the management of SSCHN. In particular, frequently they do not have all the needed professional skills, and some of them do not have a decisional network to take care of patients. In other cases, the centers or the professionals involved have a recent and unconsolidated experience in this specific field of oncological care. However, this information cannot be considered statistically significant and confirmation is needed from an even greater number of clinicians.

## 5. Conclusions

The results of the present survey confirm that oncology physicians tend to have a good perception of the communication skills of their colleagues, and they are generally satisfied with their work in relation to communication with patients. Several improvements in H&N cancer treatment recall a greater expectation of collaborative decision making, with professionals and patients participating as partners to achieve an agreement on goals in accordance with personal beliefs, values, and attitudes.

The survey, although it involved a limited number of specialists, highlighted that there is a need for greater communication both by the patient and by the doctor. In this field, the need to have specific training to improve the level of empathy is clear. The participants underlined how clinical practice and excessive work take away precious time from honest and constructive communication. Lack of staff and lack of time still represent barriers not only for a multidisciplinary approach but also for communication. This survey confirmed that although head and neck doctors will spend decades in medical education and advanced training to learn interventions, communication skills and instructional sessions are generally not considered of similar value.

**Figure 1:**
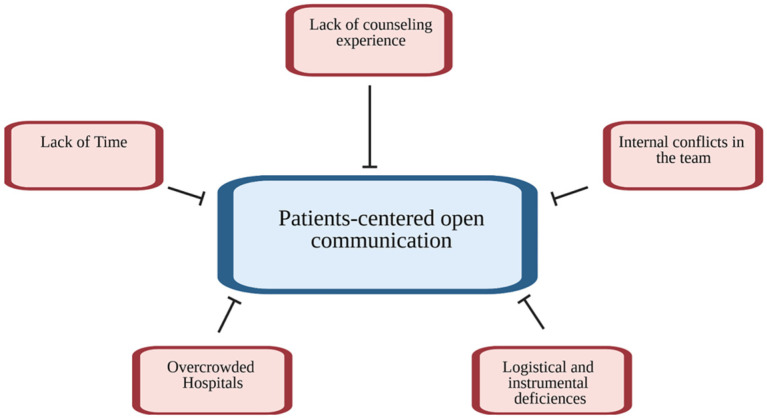
Major barriers for patient-centered care.

When talking about critical topics, such as advanced cancer, therapeutic options including immunotherapy or radiation therapy, and possible end-of-life decisions, patients want doctors to tell them honestly about their condition. Actual obstacles limit communication and make these decisions more difficult and often incomprehensible. However, the present survey showed that a frank and complete communication between doctor and patient continues to be hindered by various factors, only partially related to clinics (Figure 1).

For most of the clinicians involved, open communication objectively represented a purpose to be achieved from now on, avoiding the existing logistic and organizational barriers. This confirms the request for training and professional updates aiming to improve the relationship with patients to better define their expectations and legitimate requests. This request was clear from the answers to question 12, where all of the participants asked for more training and updating on counseling and effective communication.

**Figure 2:**
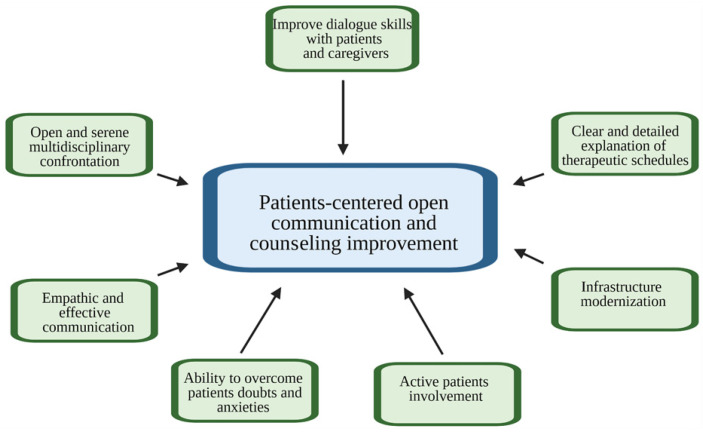
Objectives to be chased to improve doctor–patient counseling.

Several topical discussions must be improved with the sole goal of ensuring effective patient-centered communication. (Figure 2). Some of these elements are obtained only through complete communication between doctor and patient.

The results of the questionnaires indicate that the lack of time represents a serious obstacle to the treatment of patients with head and neck cancer. Therefore, there is a need to implement solutions for the medical personnel involved in the treatment program for those patients in Southern Italy. The data confirm the importance of multidisciplinary confrontation and suggest the implementation of clear communication training with the stated aim of involving both the patient and their family in therapeutic choices.

This survey underlined how clinicians clearly understand the importance of involving the patient and their family in the treatment path with an active and leading role. To achieve this, the doctor cannot ignore the role of counseling for a clear and complete communication.

## Figures and Tables

**Table 1:** The questionnaire proposed to clinicians involved in the treatment of patients with metastatic head and neck cancer.

Surname (Initial)			Name (initial):							
Age:			Sex:			Specialization:				
Years of practice in SSCHN treatment										
Total number and specialization of HCP involved in patient treatment:										
To complete this survey, order by priority all the statements below, from 1 to 5, where 1 stays for “the most relevant” and 5 for “the least relevant”.										
Please, leave out the statement not relevant according to your experience.										
1. Which are the foundations that strengthen a therapeutical alliance?										
							Value			
	Informed consent									
	Clear and complete communication									
	Organized and efficient healthcare personnel									
	Comfortable care environment									
	Welcoming and helpful staff									
	Others: ................................................................... ..............................................................................									
2. Which do you think are the aspects that make communication between patient and health care practitioner (HCP) stronger and more efficient?										
							Value			
	Investigate patient and caregivers’ expectations for the current visit, and face every point during the examination									
	Give an exhaustive form with all the specific for therapy									
	Constantly monitor the patient’s and caregiver’s level of understanding									
	Pay attention to how messages are received by the patient and the caregiver, and consequently modulate the subsequent communication									
	Collect relevant information about patient history and lifestyle, to adapt/integrate therapy in patient’s daily life									
	Others: ................................................................... ..............................................................................									
3. Which do you consider the better strategy to implement a care program focused on patient and patient family?										
							Value			
	Anticipate patient need and be proactive in care program organization									
	Train nursing staff to provide to the patient all the required informations after medical examination									
	Consider patient’s convenience and availability of resources when prescribing exams									
	Use a multidisciplinary approach to reduce waiting list									
	Adequately inform about all the successive steps and waiting times, to reduce patient and caregiver anxiety									
	Have an efficient and organized unit									
	Others: ................................................................... ..............................................................................									
4. Which competence would you like to improve?										
							Value			
	Technical skills									
	Dialogue and patient management									
	Ability to communicate openly with colleagues									
	Management skills									
	Pharmaco-economy skills									
	Others: ................................................................... ..............................................................................									
5. Which of the following do you think is an obstacle to a patient-focused care?										
							Value			
	Focus on therapy details and not to patient daily life and routine									
	Daily amount of work									
	Scientific and clinical skills									
	Infrastructure shortage									
	Access to care									
	Others: ................................................................... ..............................................................................									
6. What inhibits empathic communication with patients in daily practice?										
							Value			
	Staff and equipment shortage									
	Lack of information for population									
	Lack of counseling knowledge and skills									
	Lack of time									
	Structure inadequacy									
	Others: ................................................................... ..............................................................................									
7. Which is the strength of your unit to pursue a patient-focused care?										
							Value			
	Quick access									
	Adequate equipment and environment									
	Multidisciplinary									
	Patient involvement in care path									
	Professional know-how									
	Others: ................................................................... ..............................................................................									
8. Which is the weakness of your unit to pursue a patient-focused care?										
							Value			
	Lack of time									
	Overcrowding									
	Inadequate equipment and environment									
	Internal conflicts									
	Lack of knowledge about counseling									
	Others:................................................................... ..............................................................................									
9. Which of the following points is the most disregarded?										
							Value			
	Ability to investigate about patient doubts and anxieties									
	Comfortable environment of care									
	Open and complete communication									
	Clear and detailed explanation of therapeutic schedules									
	Patient involvement									
	Others:................................................................... ..............................................................................									
10. Which aspect would you like to improve?										
							Value			
	Open multidisciplinary confrontation									
	Dialogue skills with patient and caregivers									
	Care and diagnostic protocols									
	Management skills									
	Environment improvement									
	Specialized nursing staff									
	Others:................................................................... ..............................................................................									
11. Which of these needs is the most relevant for metastatic and/or recurrent head and neck squamous cell carcinoma (SCCHN) patients?										
							Value			
	Nutritional counseling									
	Pain therapy									
	Depression and anxiety									
	Open communication									
	Therapy side effects management									
	Talk about disease impact in patient life									
	Others:................................................................... ..............................................................................									
12. Which Continuing Medical Education (ECM) updating would you more need or prefer?										
							Value			
	Immunotherapy									
	Radiotherapy specific topics									
	Pharmacoeconomy									
	Counseling and effective communication									
	Legal issues									
	Palliative care									
	Nutrition									
	Others:................................................................... ..............................................................................									
13. Which is the more relevant topic in head and neck squamous cell carcinoma (SSCHN) therapy?										
							Value			
	Multidisciplinary									
	Treatment protocols specific for pathology									
	Counseling and effective communication									
	Frail patient									
	Palliative care									
	Nutrition									
	Others:................................................................... ..............................................................................									
14. From 1 to 10, how much are you using counseling techniques in your daily clinical practice?										
1	2	3	4	5	6	7	8	9	10	
15. Give a short explanation to the score of question 14 ............................................................................................................................. ............................................................................................................................. .............................................................................................................................										
16. Shortly describe what will help you to improve the score of question 14 ............................................................................................................................. ............................................................................................................................. .............................................................................................................................										
17. Thinking to your daily work, you will describe yourself in respect to the obstacles in counseling with the patient.										
							Value			
	Deeply unsatisfied									
	Fairly unsatisfied									
	Partially satisfied									
	Satisfied									
	Very satisfied									
Why? ............................................................................................................................. ............................................................................................................................. .............................................................................................................................										

**Table 2:** Distribution of the responses to question seven provided by the clinicians, oncologists, and radiotherapists involved in the online survey.

Which is the Strength of Your Unit to Pursue a Patient-Focused Care?	Value (*n* = 19)
*Quick access*	6
*Adequate equipment and environment*	4
*Multidisciplinary*	3
*Patient involvement in care path*	5
*Professional know-how*	1
*Others*	

**Table 3:** Distribution of the responses to question four provided by the clinicians, oncologists, and radiotherapists involved in the online survey.

Which Competence Would You Like to Improve	Value (*n* = 19)
*Technical skills*	2
*Dialogue and patient management*	9
*Ability to communicate openly with colleagues*	3
*Management skills*	2
*Pharmacoeconomy skills*	2
*Others*	

## Data Availability

All data generated or analyzed during this study are included in this published article.
